# Age-Related Changes in the Plasticity of Neural Networks Assessed by Transcranial Magnetic Stimulation With Electromyography: A Systematic Review and Meta-Analysis

**DOI:** 10.3389/fncel.2019.00469

**Published:** 2019-10-24

**Authors:** Xiaorong Tang, Peidong Huang, Yitong Li, Juanchao Lan, Zhonghua Yang, Mindong Xu, Wei Yi, Liming Lu, Lin Wang, Nenggui Xu

**Affiliations:** ^1^Medical College of Acu-Moxi and Rehabilitation, Guangzhou University of Chinese Medicine, Guangzhou, China; ^2^Acupuncture and Massage Rehabilitation Institute, Yunnan University of Chinese Medicine, Kunming, China; ^3^Clinical Research Center, South China Research Center for Acupuncture and Moxibustion, Medical College of Acu-Moxi and Rehabilitation, Guangzhou University of Chinese Medicine, Guangzhou, China

**Keywords:** plasticity of neural networks, age-related, transcranial magnetic stimulation, resting motor threshold, meta-analysis

## Abstract

**Objective:** The excitability of cerebral cortical cells, neural pathway, and neural networks, as well as their plasticity, are key to our exploration of age-related changes in brain structure and function. The combination of transcranial magnetic stimulation (TMS) with electromyography (EMG) can be applied to the primary motor cortex; it activates the underlying neural group and passes through the corticospinal pathway, which can be quantified using EMG. This meta-analysis aimed to analyze changes in cortical excitability and plasticity in healthy elderly individuals vs. young individuals through TMS-EMG.

**Methods:** The Cochrane Library, Medline, and EMBASE databases were searched to identify eligible trials published from database inception to June 3, 2019. The Cochrane Risk of Bias Tool and improved Jadad scale were used to assess the methodological quality. A meta-analysis of the comparative effects was conducted using the Review Manager 5.3 software and Stata 14.0 software.

**Results:** The pooled results revealed that the resting motor threshold values in the elderly group were markedly higher than those reported in the young group (mean difference [MD]: −2.35; 95% confidence interval [CI]: −3.69 to −1.02]; *p* < (0.00001). The motor evoked potential amplitude significantly reduced in the elderly group vs. the young group (MD: 0.18; 95% CI: 0.09–0.27; *p* < 0.0001). Moreover, there was significantly longer motor evoked potential latency in the elderly group (MD: −1.07; 95% CI: −1.77 to −0.37]; *p* =(0.003). There was no significant difference observed in the active motor threshold between the elderly and young groups (MD: −1.52; 95% CI: −3.47 to −0.42]; *p* =(0.13). Meanwhile, only two studies reported the absence of adverse events.

**Conclusion:** We found that the excitability of the cerebral cortex declined in elderly individuals vs. young individuals. The findings of the present analysis should be considered with caution owing to the methodological limitations in the included trials. Additional high-quality studies are warranted to validate our findings.

## Introduction

As the birth rate decreases and life expectancy increases, the aging problem of the global population becomes more prominent (Tatti et al., 2016). One of the most striking features of human motor behavior is the ability to respond rapidly and appropriately to environmental changes. However, this ability gradually declines with age. In particular, the decline in cognitive and motor abilities is associated with advancing age (Hunter et al., 2001). This age-related decline is a precursor of various diseases. An enhanced understanding of the impact of aging on cortical functioning may provide us more insight into understanding of age-related diseases (Seidler et al., 2010).

The human motor cortex is capable of undergoing persistent morphological or functional changes depending on stimuli from the environment; this is termed neuroplasticity (Boroojerdi et al., 2001). Cortical plasticity decreases with extensive changes in neurochemistry and neurophysiology during physiological aging (Brunso-Bechtold et al., 2000; Rossini et al., 2007). Although the regulatory mechanisms of related brain neurons are unclear, an increasing body of evidence suggests that aging is associated with alterations in the neural projections. Normal aging is associated with impairments in dendritic morphology, loss of synaptic contact cellular connectivity, and gene expression, which subsequently cause a relative decrease in the excitability of the cortex (Godde et al., 2002; Sawaki et al., 2003; Burke and Barnes, 2006; Hortobágyi et al., 2006; Oliviero et al., 2006). This eventually leads to a decrease in sensation, motor performance, and cognitive function (Mora et al., 2007; Ward et al., 2008).

Several studies showed that the decline in cognitive, memory, and behavioral abilities in healthy elderly individuals are closely related to vascular damage and amyloid deposition (DeCarli et al., 2012). Further research indicated that ischemia and amyloid deposition are connected through the utilitarian bunch of cerebrovascular cells, supporting glial tissue and neurons. Moreover, as a potential mediator of reactive aging, amyloid deposition may lead to changes in neural networks and circuits (Iadecola, 2010). In this way, the interaction of blood vessels and neurodegenerative injuries may influence the cortical and subcortical neural systems. Importantly, this damage may alter the local field potentials. In other words, we can assess age-related cognitive decline by measuring changes in the local field potentials. Recently, neuroscience research has explored the opportunity to apply Non-Invasive Brain Stimulation technology, which measures changes in the local field potentials of the cerebral cortex, to healthy elderly populations. This approach aims to explore aging-related mechanisms and the changes of cerebral cortex under physiological conditions.

The combination of transcranial magnetic stimulation with electromyography may provide information regarding the local and global potentials of the cerebral cortex, and may be used to measure changes in cortical properties following age-related changes in the plasticity of neural networks. A few studies have investigated age-related changes in the plasticity of neural networks among young and elderly individuals (Godde et al., 2002; Sawaki et al., 2003; Oliviero et al., 2006). Owing to the small sample sizes, device characteristics, parameter settings, and experimental procedures of these studies, the current excitability of the cerebral cortex at different ages remains inconclusive. We conducted a systematic review and meta-analysis of the available data to further examine this trend. Therefore, this meta-analysis aimed to analyze changes in cortical excitability and plasticity in healthy elderly individuals vs. young individuals through TMS-EMG.

## Methods

**Figure 1:**
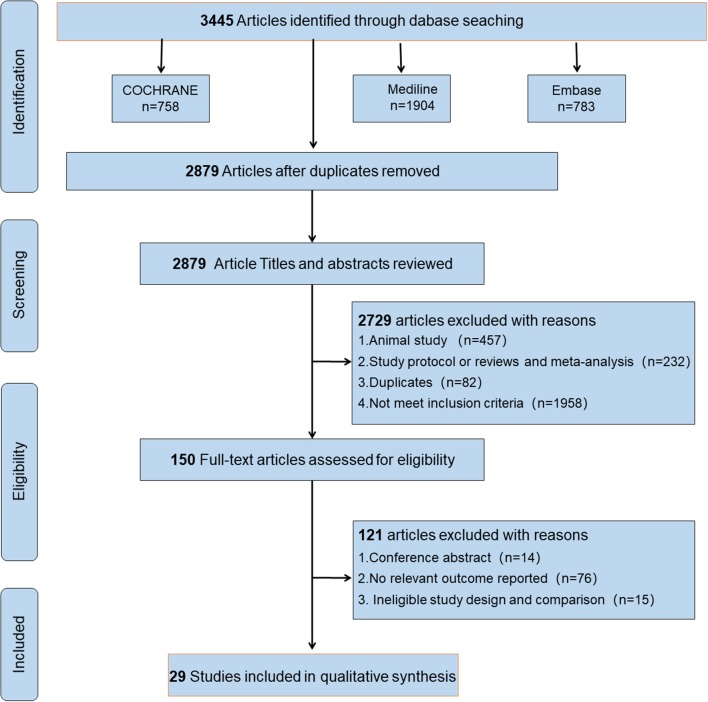
Flow diagram of studies identified, included, and excluded.

This study was performed according to the guidelines of the Preferred Reporting Items for Systematic Reviews and Meta-analysis checklist. The checklist and flowchart are shown in Figure 1, Appendix 1 in Supplementary Material.

### Search Strategy

We searched the Cochrane Library, Medline, and EMBASE databases for eligible trials published from database inception to June 3, 2019 without language restriction. The search terms are shown in Appendix 2 (Supplementary Material). In addition, we searched the websites of the international clinical trial registry provided by the U.S. National Institutes of Health to avoid missing unpublished studies. Furthermore, the references cited in the searched articles were also carefully reviewed. The search terms were listed in the Appendix 2 (Supplementary Material).

### Inclusion Criteria

(1) Patients: healthy individuals aged >18 years. (2) Interventions: all studies that used the TMS to detect brain cortical excitability and plasticity were included. (3) Comparison: healthy elderly (≥50 years) vs. healthy young (<50 years) individuals. (4) Outcomes: primary outcome measures included resting motor threshold (RMT); secondary outcome measures included any of the following: (a) motor evoked potentials (MEPs) amplitude or latency, (b) active motor threshold (AMT), and (c) adverse events. (5) Trial design: cohort or case-control studies.

### Exclusion Criteria

(1) Patients with a history of neurological or psychiatric disease, or currently receiving treatment with psychoactive drugs (i.e., sedatives, antipsychotics, antidepressants, etc.). (2) Studies with insufficient data or irrelevant outcomes.

### Study Selection and Data Extraction

All potentially eligible studies were independently selected by two reviewers (JL and YL) based on the titles and abstracts. The full text of the selected articles was subsequently obtained and independently reviewed by the reviewers based on the inclusion and exclusion criteria. The following information was independently extracted from the included trials by these two reviewers: title and authors, year of publication, sample size, age and sex of participants, target muscle and hemisphere for TMS, and outcomes (i.e., outcome measures and adverse events). Any disagreements were resolved through discussion with the third reviewer (WY) or further evaluation. The original investigators of studies with missing or incomplete data were contacted to request the data.

### Assessment of Risk of Bias (ROB)

The ROB tool of Cochrane (Savović et al., 2014) was used to assess the methodological quality. In addition, the quality of the study was evaluated using the improved Jadad scale. The full score is 7 points, and the threshold for high-quality research is ≥4 points. Two reviewers (JL and YL) independently judged the quality; any disagreements were resolved through discussion with the third reviewer (WY) acting as an arbiter. Moreover, we completed the Standards for Reporting Interventions in Controlled Trials checklist to assess the risk of bias.

### Statistical Analysis

The RevMan software (version 5.3; Cochrane Collaboration, Oxford, UK) and Stata software (version 12.0; Stata Corp LP, College Station, TX, USA) were used for statistical analysis. Dichotomous outcomes were expressed with the odds ratio and 95% confidence intervals (CI), while continuous outcomes were expressed with the mean difference (MD, indicators changed from baseline) with 95% CI. Cochran's Q test and *I*^2^ statistic were used to assess the homogeneity; the *I*^2^ test was performed for further analysis. A random-effects model was used to calculate the pooled effect size for the presence of significant heterogeneity (*p* ≤ 0.1, *I*^2^ ≥ 50%), and a fixed-effects model was applied for the absence of significant heterogeneity (Higgins et al., 2003). We explored the possible sources of heterogeneity through a subgroup analysis, and repeated the sensitivity analysis to study its effect on the overall effect size. A *p* ≤ 0.05 denoted statistically significant difference.

## Results

### Study Selection

We initially retrieved 3,445 publications, and 566 duplicate citations were removed. After screening the abstracts and titles, a total of 2,729 publications were excluded. A total of 121 publications from the 150 publications further identified as potentially eligible trials according to the review of the full text were excluded for the following reasons: 14 were conference abstracts, 76 did not report a relevant outcome, and 15 had ineligible study design and comparison. We did not identify any other studies for evaluation after reviewing the bibliographies of the full-text articles collected during the initial search. Finally, 29 studies (Rossini et al., 1992; Kossev et al., 2002; Sale and Semmler, 2005; Hortobágyi et al., 2006; Fujiyama et al., 2009, 2011, 2012, 2014; Pellicciari et al., 2009; Rogasch et al., 2009; Smith et al., 2009, 2011; Cirillo et al., 2010, 2011; Fathi et al., 2010; Todd et al., 2010; Degardin et al., 2011; Levin et al., 2011; Bernard and Seidler, 2012; Young-Bernier et al., 2012, 2014; Cuypers et al., 2013; Bashir et al., 2014; Opie and Semmler, 2014; Dickins et al., 2015, 2017; Opie et al., 2017, 2018; Emonson et al., 2019) fulfilled our inclusion criteria and were selected. Figure 1 depicts the search process and trial selection.

### Description of Included Studies

The studies included in the analysis involved a total of 914 participants. The age ranged 18–84 years. The brain cortical excitability and plasticity were examined by TMS-EMG in these studies. The muscles which evoked motor potentials in these studies included the first dorsal interosseous muscle, the abductor pollicis brevis muscle, the extensor carpi radialis muscle, the flexor carpi radialis muscle, the flexor pollicis brevis muscle, and the thenar and plantar muscles. The outcomes used to assess cortical excitability and plasticity included RMT, AMT, MEP amplitude, and MEP latency. The detailed characteristics are shown in Table 1.

**Table 1:** Characteristics of trials included in this review.

**References**	**Region**	**Participants (Y/O)**	**Age Y (years)**	**Age O (years)**	**Gender T (F/M)**	**Gender C (F/M)**	**Muscle**	**Hemisphere**	**Outcome indicator**	**Risk of bias**
Rossini et al., 1992	Italy	25/40	25.3 ± 5.3	66.0 ± 10.4	15/10	26/14	TP	All	RMT\MEP Amplitude\MEP Latency	UUUULLU
Kossev et al., 2002	Germany	10/10	28.5 ± 5.2	56.1 ± 4.9	6/4	7/3	ECR	Left	RMT\MEP Amplitude\MEP Latency	UUUULHU
Sale and Semmler, 2005	Australia	10/10	26.6 ± 1.3	67.6 ± 2.3	5/5	5/5	FDI	Left	RMT\MEP Amplitude	UUUULLU
Hortobágyi et al., 2006	USA	6/6	27.2 ± 3.7	72.7 ± 6.3	4/2	5/1	FCR	Left	RMT\MEP Amplitude	UUUULLU
Rogasch et al., 2009	Australia	14/14	20.7 ± 1.9	68.3 ± 5.6	6/8	6/8	APB	Left	RMT\AMT\MEP Amplitude	UUUULLU
Pellicciari et al., 2009	Italy	16/16	26.2 ± 0.8	62.1 ± 1.5	8/8	8/8	APB	Left	RMT\MEP Latency	UUUULLU
Fujiyama et al., 2009	Australia	15/15	18–33	61–75	9/6	9/6	ECR	Left	MEP Amplitude	UUUULLU
Smith et al., 2009	Australia	13/17	20.0 ± 2.0	63.1 ± 4.2	0/13	0/17	FDI	Left	RMT\MEP Amplitude\AMT	UUUULLU
Fathi et al., 2010	Japan	16/16	21–39	60–79	2/14	5/11	APB	Left	RMT\MEP	UULLLLU
Cirillo et al., 2010	Australia	12/14	22 ± 2	67 ± 4	7/5	7/7	APB	Left	RMT\AMT\MEP Amplitude	UUUULLU
Todd et al., 2010	Australia	15/15	25 ± 4	67 ± 5	6/9	6/9	FDI	Left	RMT\MEP\AMT	UULULLU
Cirillo et al., 2011	Australia	16/16	23 ± 3	67 ± 5	9/7	9/7	FDI	Left	RMT\AMT\MEP Amplitude	UUULLLU
Degardin et al., 2011	France	14/14	26.4 ± 2.7	62.4 ± 7.1	6/8	8/6	APB	N/A	RMT\AMT	UUUULLU
Fujiyama et al., 2011	Australia	13/13	18–34	62–74	9/4	9/4	FPB	Left	RMT	UULULLU
Levin et al., 2011	Belgium	6/5	23.7 ± 2.3	63.8 ± 1.8	N/A	N/A	APB	Left	RMT\MEP Amplitude	UUUULLU
Smith et al., 2011	Australia	15/15	20.1 ± 2.1	65.5 ± 3.9	0/15	0/15	FDI	Left	RMT\AMT	UUUULLU
Bernard and Seidler, 2012	USA	16/17	21 ± 1.83	69.53 ± 4.07	12/4	10/7	FDI	Left	RMT\MEP Amplitude\MEP Latency	UUUULLU
Fujiyama et al., 2012	Australia	15/15	18–29	58–84	8/7	9/6	ECR	Left	RMT\MEP Amplitude	UUULLLU
Young-Bernier et al., 2012	Canada	25/31	22.5 ± 3.5	70.3 ± 3.8	14/11	18/13	FDI	Left	RMT\MEP Amplitude\MEP Latency	UULULLU
Cuypers et al., 2013	Belgium	14/10	22.8 ± 1.7	69.3 ± 2.8	8/6	8/2	FDI	Left	RMT\MEP Amplitude	UUUULLU
Bashir et al., 2014	USA	10/8	23.40 ± 3.50	57.38 ± 9.61	4/6	5/3	FDI	Left	RMT\MEP Amplitude	UUUULLU
Fujiyama et al., 2014	Australia	20/20	22.7 ± 3.3	68.3 ± 7.9	13/7	10/10	FCR	Left	RMT	UULLLLU
Opie and Semmler, 2014	Australia	22/18	22.3 ± 3.1	70.8 ± 5.0	N/A	N/A	FDI	N/A	RMT\AMT\MEP Amplitude	UULLLLU
Young-Bernier et al., 2014	Canada	20/18	22.3 ± 3.2	70.1 ± 5.6	13/7	9/9	FDI	N/A	RMT\MEP Amplitude\MEP Latency	UULULLU
Dickins et al., 2015	Australia	20/20	22.95 ± 2.52	70.15 ± 3.07	10/10	10/10	APB	N/A	RMT\MEP	UULULLU
Dickins et al., 2017	Australia	20/20	24.4 ± 3.86	69.55 ± 3.99	10/10	10/10	APB	N/A	RMT\MEP	UULLLLU
Opie et al., 2017	Australia	15/15	22.9 ± 0.5	70.8 ± 1.6	8/7	7/8	FDI	Left	RMT\MEP	UUULLLU
Opie et al., 2018	Australia	15/18	22.5 ± 2.9	70.1 ± 6.0	8/7	12/6	FDI	Left	RMT\MEP\AMT	UUULLLU
Emonson et al., 2019	Australia	20/20	24.5 ± 4.48	65.47 ± 5.62	N/A	N/A	N/A	N/A	RMT	UUULLLU

*Y, young group; O, old group; F, female; M, male; N/A, not applicable; TP, thenar and plantar muscles; ECR, the extensor carpi radialis muscle; APB, the abductor pollicis brevis muscle; FDI, the first dorsal interosseous muscle; FCR, the flexor carpi radialis muscles; FPB, the flexor pollicis brevis muscle*.

### Methodological Quality of Included Studies

**Figure 2:**
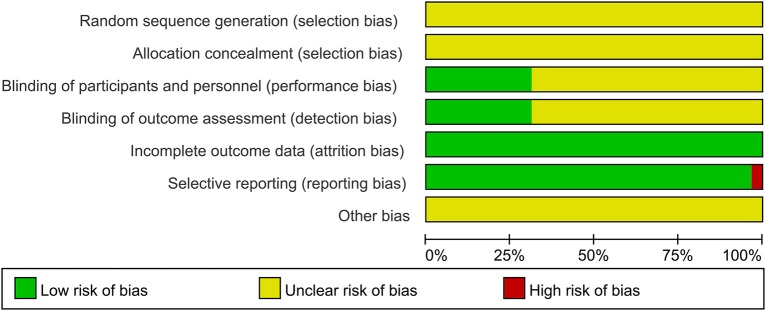
Graph of the risk of bias in the included trials by Cochrane risk of bias toll based upon reviewers' judgment of each domain.

**Figure 3:**
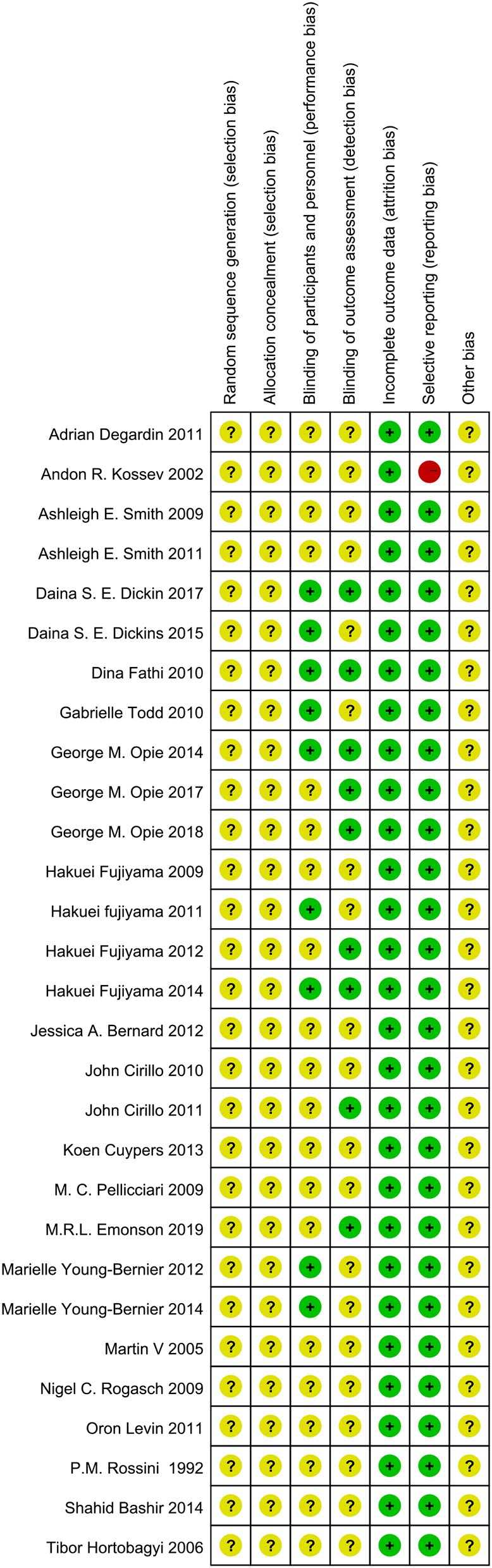
Summary Graph of the risk of bias in the included trials by Cochrane risk of bias toll based upon reviewers' judgment of each domain.

Randomization was not used in the 29 selected studies, and none of the studies described concealment of allocation. Nine studies (Fathi et al., 2010; Todd et al., 2010; Fujiyama et al., 2011, 2014; Young-Bernier et al., 2012, 2014; Opie and Semmler, 2014; Dickins et al., 2015, 2017) used appropriate blinding methods for the participants, and nine studies (Fathi et al., 2010; Cirillo et al., 2011; Fujiyama et al., 2012, 2014; Opie and Semmler, 2014; Dickins et al., 2017; Opie et al., 2017, 2018; Emonson et al., 2019) blinded the outcome assessments. All studies reported complete outcome data. Overall, the methodological quality all the included studies was low. The details are listed in the Appendix 3 (Supplementary Material). The summary of bias in each domain across the included studies is shown in Figures 2, 3.

### Primary Outcomes

**Figure 4:**
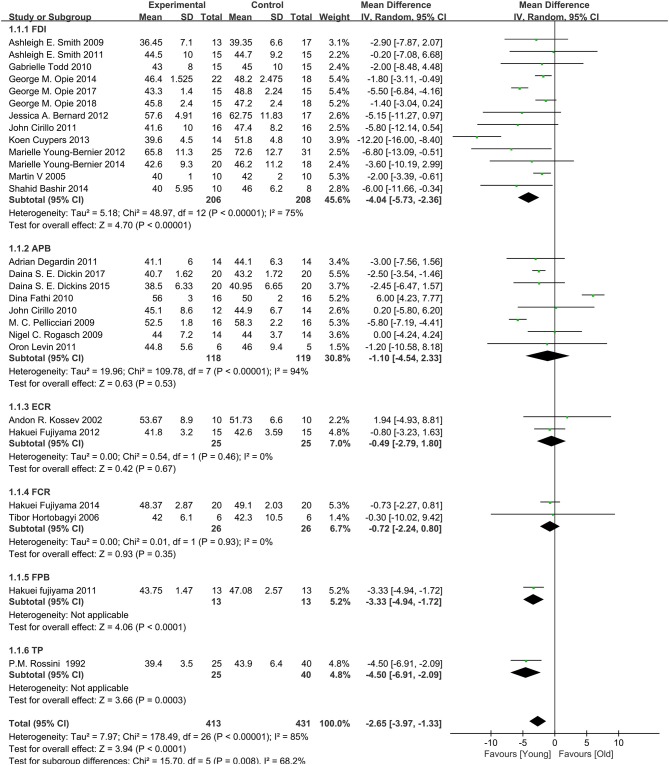
Young group vs. old group, RMT.

A total of 28 studies (Rossini et al., 1992; Kossev et al., 2002; Sale and Semmler, 2005; Hortobágyi et al., 2006; Pellicciari et al., 2009; Rogasch et al., 2009; Smith et al., 2009, 2011; Cirillo et al., 2010, 2011; Fathi et al., 2010; Todd et al., 2010; Degardin et al., 2011; Fujiyama et al., 2011, 2012, 2014; Levin et al., 2011; Bernard and Seidler, 2012; Young-Bernier et al., 2012, 2014; Cuypers et al., 2013; Bashir et al., 2014; Opie and Semmler, 2014; Dickins et al., 2015, 2017; Opie et al., 2017, 2018; Emonson et al., 2019) assessed the changes in RMT between the elderly and young groups (Figure 4). Owing to the significant heterogeneity among the studies (*I*^2^: 85%; *p* < 0.00001), we selected the random-effects model. The pooled results revealed that the RMT values in the elderly group were markedly higher than those observed in the young group (MD: −2.65; 95% CI: −3.97 to −1.33; *p* = 0.008).

#### Subgroup Analysis of Primary Outcomes

Owing to the high heterogeneity among the pooled results of RMT evaluation (*I*^2^ = 85%; *p* < 0.00001), we divided the studies according to the year of publication and region, and the varied stimulated muscles were examined through a subgroup analysis (Table 2, Figure 4, Appendices 4, 5 in Supplementary Material). The subgroup analysis showed that the muscle, region, or year of publication may be the sources of high heterogeneity.

**Table 2:** Subgroup analysis based on the RMT.

**Group**	**No. of studies**	**No. of participants**	***MD***	**95% *CI***	***Z***	***p* (Effect)**	***I^2^* (%)**	**χ^2^**
**Muscle**								
FDI	13	414	−4.04	[−5.73, −2.36]	4.70	<0.00001	75	48.97
APB	8	237	−1.10	[−4.54, 2.33]	0.63	0.53	94	109.78
ECR	2	50	−0.49	[−2.79, 1.80]	0.42	0.67	0	0.54
FCR	2	52	−0.72	[−2.24, 0.80]	0.93	0.35	0	0.01
FPB	1	26	−3.33	[−4.94, −1.72]	4.06	<0.00001		
TP	1	65	−4.50	[−6.91, −2.09]	3.66	0.0003		
**Publication region**								
Oceania	16	515	−1.90	[−2.95, −0.85]	3.56	0.0004	69	48.82
Europe	6	180	−5.10	[−7.95, −2.25]	3.51	0.0005	74	19.21
Asia	1	32	6	[4.23, 7.77]	6.66	<0.00001		
America	5	157	−4.99	[−7.91, −2.06]	3.34	0.0008	0	1.51
**Publication year**								
≤ 2011	15	422	−1.65	[−3.97, 0.68]	1.38	0.17	88	118.78
>2011	13	462	−2.94	[−4.55, −1.33]	9.35	0.0004	84	73.13

*ECR, the extensor carpi radialis muscle; APB, the abductor pollicis brevis muscle; FDI, the first dorsal interosseous muscle; FCR, the flexor carpi radialis muscles; FPB, the flexor pollicis brevis muscle; TP, thenar and plantar muscles*.

#### Subgroup Analysis Based on Different Stimulated Muscles

The pooled results showed that the excitability of the cerebral cortex in the elderly group was significantly reduced compared with that reported in the young group. However, subgroup differences were detected, and when the stimulated muscle was the abductor pollicis brevis muscle (MD: −1.10; 95% CI: −4.54–2.33; *p* = 0.53), extensor carpi radialis (MD: −0.49; 95% CI: −2.79–1.80; *p* = 0.67), and flexor carpi radialis (MD: −0.72; 95% CI: −2.24–0.80; *p* = 0.35), there was no significant difference between two groups (Figure 4). This indicates that the measurement effects of different muscles vary. Therefore, different muscles may be representative of high heterogeneity.

#### Subgroup Analysis Based on Different Regions

We divided the regions of study into Oceania, Europe, Asia, and America. The results showed that the excitability of the cerebral cortex in the elderly group was significantly reduced compared with that recorded in the young group. However, one study in Asia showed a decrease in RMT in the control group (Appendix 4 in Supplementary Material). It is possible that the statistical power was inadequate due to the small number of studies performed in Asia.

#### Subgroup Analysis Based on the Year of Publication

We divided the studies according to the year of publication (i.e., in and prior to 2011, and after 2011). The results showed that the excitability of the cerebral cortex in the elderly group was markedly lower than that reported in the young group. However, we did not find significant differences in the two groups among the studies performed in and prior to 2011 (MD: −1.65; 95% CI: −3.97–0.68; *p* = 0.17) (Appendix 5 in Supplementary Material). This may be related to device characteristics (e.g., type of coil and accuracy of the EMG) or the level of operational proficiency.

#### Sensitivity Analysis

**Figure 5:**
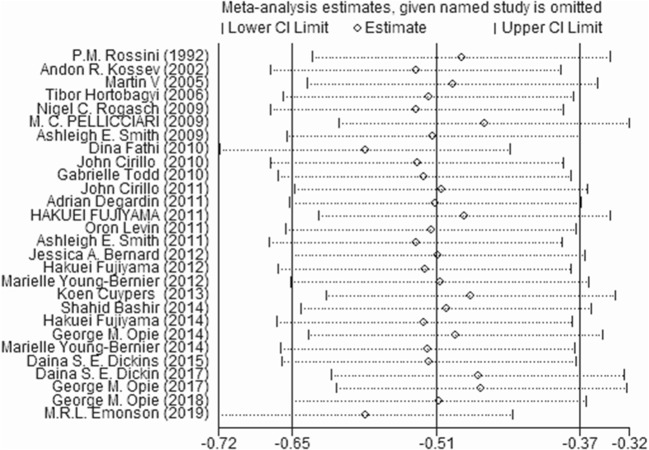
Sensitivity analysis by excluding each article.

We excluded one study at a time to determine the effect of each study on the estimated pooled effect size. The results did not show instability (Figure 5).

### Secondary Outcomes

#### MEP Amplitude

**Figure 6:**
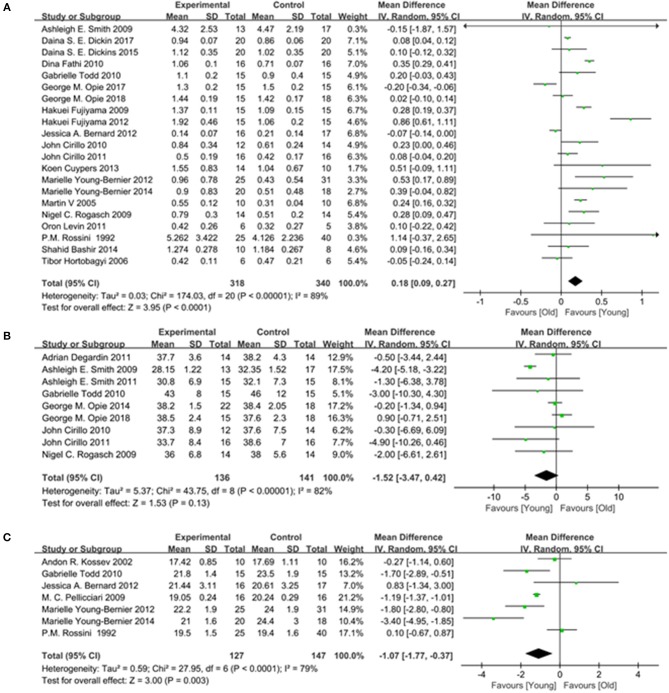
Young group vs. old group, Secondary outcomes. **(A)** Forest plot of the MEP amplitude. **(B)** Forest plot of the MEP Latency. **(C)** Forest plot of the AMT.

A total of 20 studies (Rossini et al., 1992; Sale and Semmler, 2005; Fujiyama et al., 2009, 2012; Rogasch et al., 2009; Smith et al., 2009; Cirillo et al., 2010, 2011; Fathi et al., 2010; Todd et al., 2010; Levin et al., 2011; Bernard and Seidler, 2012; Young-Bernier et al., 2012, 2014; Cuypers et al., 2013; Bashir et al., 2014; Dickins et al., 2015, 2017; Opie et al., 2017, 2018) providing numerical data for the MEP amplitude were included. Owing to the heterogeneity, a random-effects model was applied (*I*^2^: 89%; *p* < 0.00001). The pooled data analysis showed that the MEP amplitude was markedly reduced in the elderly group vs. the young group (MD: 0.18; 95% CI: 0.09–0.27; *p* < 0.00001) (Figure 6A).

#### MEP Latency

Seven studies (Rossini et al., 1992; Kossev et al., 2002; Pellicciari et al., 2009; Todd et al., 2010; Bernard and Seidler, 2012; Young-Bernier et al., 2012, 2014) providing numerical data for the MEP latency were included. Owing to the heterogeneity, a random-effects model was used (*I*^2^: 79%; *p* < 0.00001). The pooled results showed that there was a significantly longer MEP latency in the elderly group (MD: −1.07; 95% CI:−1.77 to −0.37; *p* = 0.003) (Figure 6B).

#### AMT

Nine studies (Rogasch et al., 2009; Smith et al., 2009, 2011; Cirillo et al., 2010, 2011; Todd et al., 2010; Degardin et al., 2011; Opie and Semmler, 2014; Opie et al., 2018) providing numerical data for the AMT were included. Owing to the heterogeneity, a random-effects model was used (*I*^2^: 82%; *p* < 0.00001). There was no significant difference observed in the AMT between the two groups (MD: −1.52; 95% CI: −3.47 to −0.42; *p* = 0.13) (Figure 6C).

### Adverse Outcomes

Only two (Hortobágyi et al., 2006; Opie et al., 2017) studies did not report adverse events related to TMS. The remaining studies did not report adverse outcomes.

### Publication Bias

According to Egger's test, there was no evidence of publication bias among the primary outcome (RMT outcome, Egger's test *p* = 0.257).

## Discussion

### Summary of the Main Findings

We conducted a meta-analysis including 29 studies with 914 patients. We used TMS-EMG, including RMT, AMT, MEP amplitude, and MEP latency to evaluate the age-related changes in the plasticity of neural networks among young and elderly individuals. The results indicated that the excitability of the cerebral cortex declined in elderly individuals vs. young individuals. Three important points were noted. Firstly, the RMT and MEP latency in the elderly group was higher than that in the young group. In contrast, the MEP amplitude in the elderly group was lower than that in the young group. Secondly, there was no significant difference in the AMT between the two groups. Third, there were no common adverse reactions reported in the included studies, suggesting that TMS-EMG technology is relatively safe.

### Age-Related Changes in the Plasticity of Neural Networks

Driven by a decline in birth rates and an increase in life expectancy, the global demographic structure is rapidly aging. Demographic projections show that the proportion of individuals aged ≥65 years is expected to increase by >150% in 35 years (Tatti et al., 2016). According to this projection, the number of individuals aged >65 years will exceed 1.6 billion by 2050. Age-related diseases profoundly impact the daily activities and quality of life of the elderly (Logsdon et al., 2002; Craik and Bialystok, 2006), posing a great and urgent challenge to society (Ballard, 2010). Therefore, it is essential to fully understand the mechanisms involved in aging.

Aging is associated with functional decline in numerous cognitive areas, such as attention, memory, language, and executive functions (Morrison and Baxter, 2012). The structure and function of many brain regions undergo extensive changes with aging, including reduction in cerebral cortex thickness (Tatti et al., 2016), decreases in neurotransmitter binding potential, and synaptic receptor density and efficacy (Celsis, 2000), change in cortical and cerebellar metabolism (Dukart et al., 2013), gray matter atrophy, white matter loss ventricular enlargement, etc. (Scahill et al., 2003; Bolandzadeh et al., 2012; Bennett and Madden, 2014).

Research on the mechanism of aging and the related physiological and pathological changes is insufficient. However, in recent years, numerous studies investigating age-related changes in the plasticity of neural networks have achieved breakthroughs in this field. A number of studies have shown that elderly individuals with normal cognition mainly showed functional changes in the neural network during memory coding, especially in the posterior medial cortex (Wellman and Sengelaub, 1995; Ahmed et al., 2018). Converging evidence from positron emission tomography studies indicates that nearly one-third of clinically normal elderly individuals harbor fibrillar β-amyloid deposition (Dickerson et al., 2009; Sperling et al., 2009). Animal experiments confirmed that with increasing age, fibrous β-amyloid is gradually deposited, and functional changes in the default network were observed in the posterior medial cortex of rats (Becker et al., 2011; Sousa et al., 2017). These studies suggested that many of the age-related changes in neural network function may be attributed to amyloid deposition (Iadecola, 2010).

Multi-dimensional observations of changes in the neural networks of the brain may deepen our understanding of aging, and its physiological and pathological mechanisms. Non-invasive brain stimulation techniques (e.g., TMS) are used in numerous studies to detect changes in the neural networks. Recently, neuroscience research has explored the possibility of applying this technology to examine the elderly population. Early detection of changes may enable the development of new treatments, thereby increasing the likelihood of effectively preventing or delaying the occurrence of disease.

### Diagnostic Applications of TMS for the Plasticity of Neural Networks

Since its introduction by Professor Anthony Barker of the University of Sheffield in 1985 (Barker et al., 1985), TMS has been used to activate the human motor cortex, non-invasively assess the net level of cortical excitability, and determine the integrity of the central motor pathways (Tatti et al., 2016). TMS removes short and powerful magnetic pulses from the skull and generates a secondary electrical current according to the principle of electromagnetic induction of Faraday. The current generated in the cerebral cortex changes the excitability of the underlying neurons and induces neuronal firing. This ultimately results in movement of the corresponding muscles through the corticospinal, corticonuclear, callosal fiber, nerve roots, and peripheral motor pathways (Sanes and Donoghue, 2000; Müller-Dahlhaus et al., 2010). In this manner, TMS may directly reflect the excitability information of different motor cortices and functional integrity of the intracortical neuronal structures. EMG refers to the muscle bioelectrical pattern recorded using an electromyograph. It is often used to determine the functional status of peripheral nerves, neurons, neuromuscular junctions, and muscles. When TMS-EMG is applied to the monitoring of primary motor cortex excitability, it activates the underlying neural group and passes through the corticospinal pathway. This ultimately leads to a motor response to the contralateral muscle MEP, which can be quantified using EMG. The motor thresholds, the MEP amplitude, the MEP latency, and short intracortical inhibition are the most commonly applied techniques for TMS (Edwards et al., 2008). Notably, the results of these techniques are largely influenced by device characteristics, parameter settings, and experimental procedures. In addition, subject-related variables (e.g., eventual pharmacological treatments) affect the outcome indicators of TMS stimulation.

In recent years, investigations regarding the plasticity of neural networks have attracted considerable attention, and researchers use the TMS-EMG technology to evaluate the sensitivity of the motor system to plastic changes. Some studies showed that following the induction of the M1 cortical region of the brain by TMS in the elderly, their ability to stimulate excitability and stimulation-induced adaptations was limited compared with that reported in the young individuals (Dayan et al., 2013; Li et al., 2015). Consistent with the findings of the present meta-analysis, these results indicate that the function and ability of the M1 cortical region in older individuals decrease with age.

### Limitations

This meta-analysis had several limitations. Firstly, heterogeneity was detected, which reduced the reliability of the comparisons. Although all the included studies were examined using only TMS-EMG, there was also heterogeneity detected in different characteristics, parameter settings, experimental procedures, and subject-related variables (including state dependency and eventual pharmacological treatments), which may reduce the generalizability of the conclusions. Secondly, only 29 randomized controlled trials were included in this review. Moreover, limitations in the quality of research design and sample sizes affect the extrapolation and strength of this evidence. In addition, due to the limitations of the inclusion of the literature, we can only be included in the cohort and case-control studies. Therefore, it is possible that the statistical power may have been inadequate due to the small number of studies, and it is difficult to precisely evaluate the excitability of neural networks in the different groups. Thirdly, there was no report of adverse events. In the included studies, we only found limited information related to safety owing to poor reporting of adverse events. Hence, the pooled results should be treated with caution. Another limitation is that, in this review, behavioral meta-analysis results are only provided by the included TMS-EMG studies, which may lead to publication bias. This suggests that some studies without significant cortical activation results may not be published.

## Conclusions

We found that the excitability of the cerebral cortex declined in elderly individuals vs. young individuals. The findings of this review should be considered with caution owing to the methodological limitation of the included trials. Further high-quality studies are warranted to validate the present findings. It is well established that the normal process of aging is the result of degeneration and compensation of the neural network and its related conduction functions. However, the existing models of age-related changes in the brain do not cover the full spectrum of alterations (Li et al., 2015). Thus, the use of various detection methods is necessary to dynamically observe the age-related changes in neural networks. This approach may assist researchers in developing a new aging model, which reflects the age-related changes in the plasticity of neural networks.

## Data Availability Statement

The data supporting the results reported in this article may be obtained from the corresponding author upon reasonable request.

## Author Contributions

NX and PH: study concept and design. WY and ZY: data curation. YL and LW: formal analysis. YL and JL: investigation. XT: methodology. YL: resources. LW, MX, and PH: software. XT and PH: supervision and writing-original draft. NX: validation. XT: writing-review and editing. All authors read and approved to publish the final version of the manuscript.

### Conflict of Interest

The authors declare that the research was conducted in the absence of any commercial or financial relationships that could be construed as a potential conflict of interest. The handling editor declared a shared affiliation, though no other collaboration, with the author PH at the time of the review.

## Abbreviations

AMT: active motor threshold
EMG: electromyography
APB: the abductor pollicis brevis muscle
ECR: the extensor carpi radialis muscle
FDI: the first dorsal interosseous muscle
FCR: the flexor carpi radialis muscle
FPB: the flexor pollicis brevis muscle
LTP: long-term potentiation
LTD: long-term depression
NIBS: Non-Invasive Brain Stimulation
PRISMA: Preferred Reporting Items for Systematic Reviews and Meta-analysis checklist
RMT: resting motor threshold
TMS: transcranial magnetic stimulation
TP: thenar and plantar muscle
TES: transcranial electrical stimulation
MEP: motor evoked potential.
