# Supplementary material for: Age-Related Changes in the Plasticity of Neural Networks Assessed by Transcranial Magnetic Stimulation With Electromyography: A Systematic Review and Meta-Analysis
Source: Front Cell Neurosci. 2019 Oct 24;13:469. doi: 10.3389/fncel.2019.00469 (PMC6822534; doi:10.3389/fncel.2019.00469)
Supplement: Supplementary file 2 [file Table_2.docx]

Appendix 2.

*Search strategy*

(“non-invasive brain stimulation” [tiab] or “Noninvasive Cranial Nerve Neuromodulation” [tiab] or “Electrical Stimulation of the Brain” [tiab] or “Brain Stimulation” [tiab] or “Cranial nerve modulation” [tiab] or “Cranial nerve modulator” [tiab] or “transcranial magnetic stimulation” [tiab] or “TMS” [tiab] or “repetitive transcranial magnetic stimulation” [tiab] or “rTMS”) And （“Aged” [tiab] or “aging” [tiab] or “elderly “senior” [tiab] or “geriatric” [tiab] or“gerontology ” [tiab] or “Senescence” [tiab] or “frail elderly “Young” [tiab] or “youngster” [tiab] or “stripling” [tiab] or “childish” [tiab] or “youthful” [tiab] or “immature” ) And （“cortical plasticity” [tiab] or “neuronal plasticity” [tiab] or “neuroplasticity” [tiab] or “neural plasticity” ）

**PICOS form:**

| Inclusion Criteria | |
| --- | --- |
| Participants | Healthy individuals aged >18 years |
| Interventions | All studies that used the TMS to detect brain cortical excitability  and plasticity were included |
| Comparisons | Healthy elderly (≥50 years) vs. healthy young (<50 years) individuals |
| Outcomes | primary outcome: RMT; secondary outcome : MEPs ,AMT,adverse events |
| Study design | cohort or case-control studies |
