# Supplementary figures and images for: Age-Related Changes in the Plasticity of Neural Networks Assessed by Transcranial Magnetic Stimulation With Electromyography: A Systematic Review and Meta-Analysis
Source: Front Cell Neurosci. 2019 Oct 24;13:469. doi: 10.3389/fncel.2019.00469 (PMC6822534; doi:10.3389/fncel.2019.00469)

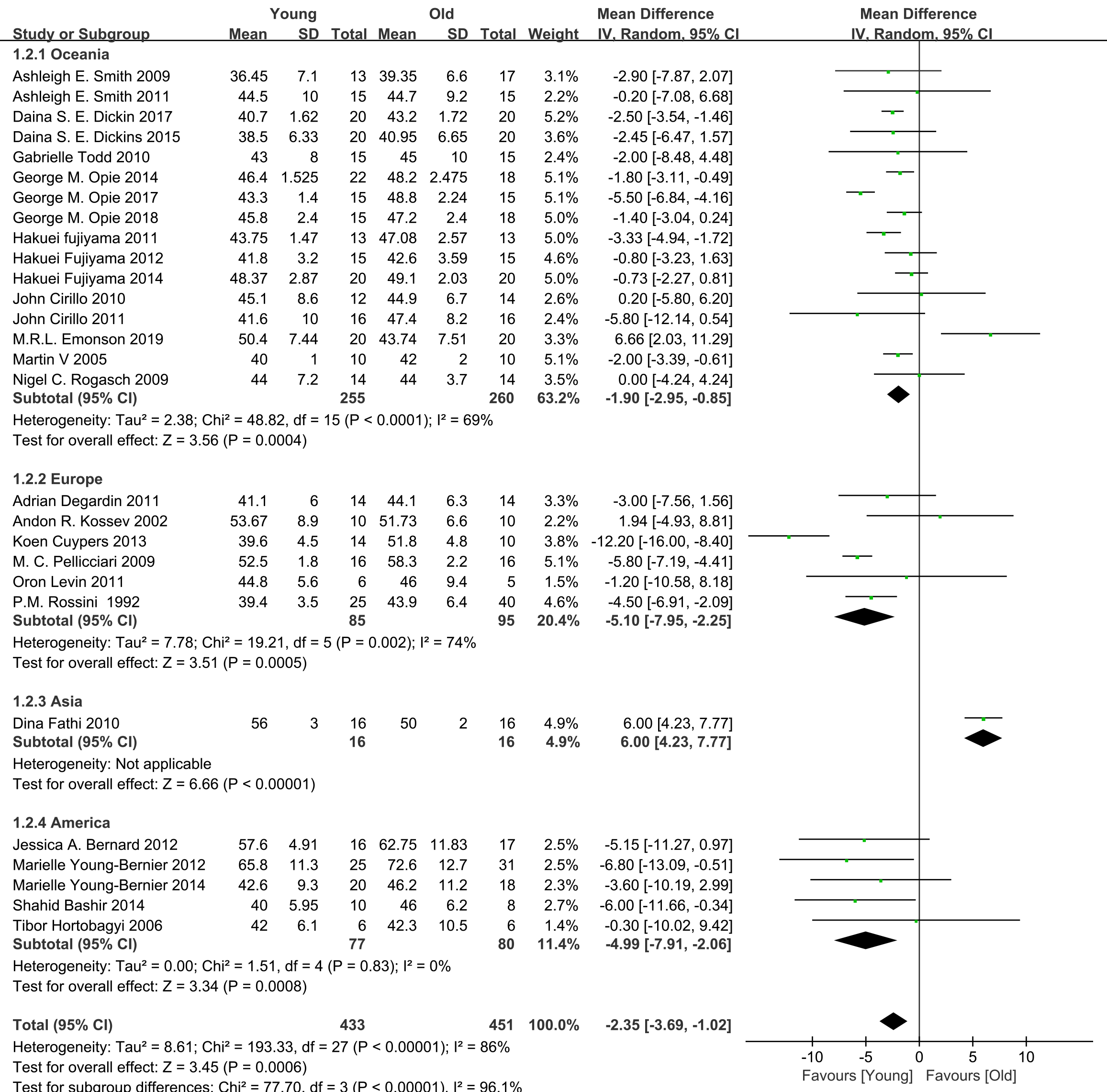

Supplement: Supplementary file 4 [file Image_1.TIF]
